# Synthesis, and evaluation of photophysical properties of a potential DPP-derived photosensitizer for photodynamic therapy with D-A-D architecture

**DOI:** 10.1007/s10856-024-06776-0

**Published:** 2024-02-01

**Authors:** Vanessa Escalona Hernández, Itzia Irene Padilla-Martínez, Rosa Angeles Vázquez García, María Aurora Veloz Rodríguez, Oscar Javier Hernández-Ortiz

**Affiliations:** 1 Área Académica de Ciencias de la Tierra y Materiales, Carretera Pachuca-Tulancingo Km, Universidad Autónoma del Estado de Hidalgo (UAEH), 4.5.C.P. 42184. Ciudad del Conocimiento, Mineral de la Reforma, Hgo México; 2 Laboratorio de Química Supramolecular y Nanociencias de la Unidad Profesional Interdisciplinaria de Biotecnología del Instituto Politécnico Nacional, Av. Acueducto s/n Barrio la laguna Ticomán, Ciudad de México, 07340 México

## Abstract

The study of a macromolecule derived from DPP and triphenylamine, (**DPP-BisTPA**) by computational chemistry, its synthesis by direct arylation, optical characterization (UV-Vis and fluorescence) and electrochemistry (cyclic voltammetry), as well as its evaluation as a generator of reactive oxygen species indirectly, through the degradation of uric acid. The results obtained by DFT using B3LYP/6-31G (d, p) and TD-DFT using CAM-B3LYP/6-31G (d, p) reveal values of energy levels of the first singlet and triplet excited state that indicate a possible intersystem crossover and the possible generation of reactive oxygen species by a type I mechanism. The compound presents an absorption region within the phototherapeutic window. The electrochemical bandgap is 1.64 eV which suggests a behavior as a semiconductor. **DPP-BisTPa** were processed as hemispherical nanoparticles with a size around 100 nm, and NPOs were evaluated as a photosensitizer with a ROS generation yield of 4% using a photodynamic therapy flashlight as the light source.

**Graphical Abstract:**

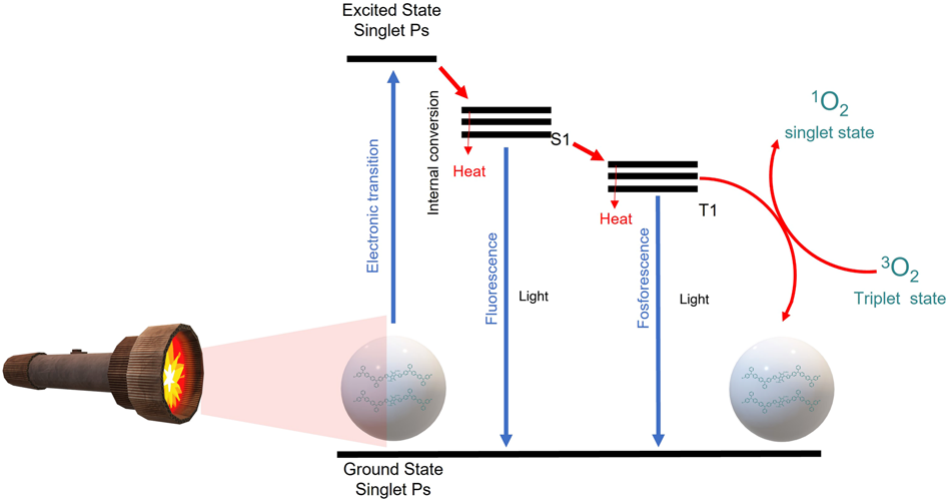

## Introduction

In recent years, materials based on organic molecules with a π-conjugated system have been developed for a wide range of applications, mainly for optoelectronics [1, 2]. However, their intrinsic properties have made them attractive for biomedical applications [3], such as their use as photosensitizing materials in photodynamic therapy [4–6], contrast agents for bioimaging [7, 8], biosensing [9–11] and theranostic materials [12–14].

Photodynamic therapy is a minimally invasive technique that has gained great popularity in recent years in treating cancer [15–17], mainly those that can be irradiated with light or use a light probe to carry out their processes [4, 15, 16]. This consists of internalizing nanomaterials called photosensitizers that are activated by light, promoting an excited state that leads to intersystem crossing (ISC) and subsequent relaxation from a triplet state that interacts with cellular molecular oxygen to generate reactive oxygen species (ROS). such as singlet oxygen (^1^O_2_), hydroxyl radicals, the superoxide anion and hydrogen peroxide and thereby promote cell apoptosis [4, 15]. In this context, this is seeking to obtain photosensitizers with an excellent intersystem crossing (ISC) rate, minimal cytotoxicity in the dark, high molar extinction coefficient, minimally between 400 and 600 nm and that absorb in the so-called phototherapeutic region that is between 600 and 800 nm [16, 17].

π-conjugated systems, either copolymers or small molecules [18–20] allow tuning of their optical properties [21–23] through a rationalized design with alternating donor (D) and acceptor (A) moieties, with D-A structures in the case of copolymers [24–26]; and in the case of small molecules, by the use of different molecular architectures [27, 28], either D-A-D [29–32] or A-D-A [33–36] favoring intramolecular charge transfer (ICT) [37, 38]. The formation of organic nanoparticles can be carried out by different methods, the most commonly used being reprecipitation, also called nanoprecipitation [12, 39–41]. Unlike inorganic nanoparticles, the properties of ONPs depend on the stacking of the molecules and their properties depend directly on the properties of the molecule they are made of [42, 43].

Diketopyrrolopyrrole (DPP) is an electron-deficient moiety with a planar π-skeleton, high molar extinction coefficient, good charge carrier mobility, large Stokes shift, good thermal-stability and photostability [44, 45]. DPP derivatives, polymers and small molecules, with excellent optical and electronic properties [46] Therefore, derivatives of DPP have been reported as materials with outstanding properties in OPVs [47, 48], OLEDs [49, 50], OFETs [51]. However, also in biomedical applications, DPP-based compounds have been reported with excellent behavior for materials for sensing [52], bioimaging [53, 54], photodynamic therapy [55, 56], and theranostics [57, 58]. DPP has shown low dark toxicity and good generation of reactive oxygen species, normally favor the generation of singlet oxygen [59]. In addition, DPP can be easily functionalized by using the 3- and/or 6-position of its ring. DPP derivatives functionalized with electron-donating groups usually show a bathochromic shift, which is of great advantage for their use in therapy [55, 60, 61]. DPP derivatives have been studied as photosensitizers in vitro using HeLa cells [62, 63] and in vivo [13, 55, 60] with excellent results, ^1^O_2_ quantum yields between 2 and 80% [57, 64, 65].

This article reports the synthesis, optical and electrochemical characterization and evaluation of the ability of a DPP-derived macromolecule with quadrupolar D-A-D architecture to generate reactive oxygen species as a potential photosensitizer material for photodynamic therapy.

## Materials and methods

The reagents 2,5-dihydro-3,6-di-2-thienyl-pyrro[3,4-c]pyrrole-1,4-dione, bromododecane, calcium hydride, 4,4′-Bis[(4- bromophenyl)phenylamino]biphenyl, palladium acetate, uric acid and cetyltrimethylammonium bromide were purchased from Sigma Aldrich™. Potassium carbonate, methylene blue, and sodium phosphate were purchased from Merk™. The solvents N,N’ dimethylacetamide and acetonitrile are anhydrous purchased from Sigma Aldrich. The THF is HPLC grade, it was purchased from J.T. Baker™. The rest of the solvents were distilled and dried by the known method.

### Synthesis

#### 2,5-didodecil-3,6-di(tiofen-2-il)-2,5-dihidropirrolo[3,4-c]pirrol-1,4-diona (DPP-Alq)

100 mg (0.333 mmol) 2,5-dihydro-3,6-di-2-thienyl-pyrro[3,4-c]pyrrole-1,4-dione, 248.9 mg (0.999 mmol) bromododecane, 50.6 mg (0.999 mmol) CaH_2_ in 10 mL dimethylformamide as solvent. The reaction mixture was kept at a temperature of 120–130 °C with stirring for 16 h. The product was precipitated by adding about 50 mL of cold methanol. The product was obtained as a dark red powder (166.3 mg, 0.2610 mmol) in 78.4% yield. Melting point: 125–128 °C. FTIR ν(cm^−1^): 3100 C-H, 2970 C-H, 1650 C=O, 1320 C-N, 705 C-S. NMR H^1^ (300 MHz, CDCl_3_) δ (ppm): 8.92 (dd, J = 3.9, 1.2 Hz, 1H), 7.64 (dd, J = 5.0, 1.2 Hz, 1H), 7.29 (dd, J = 5.0, 3.9 Hz, 1H), 4.13 – 4.02 (m, 2H), 1.74 (t, J = 7.8 Hz, 2H), 1.40 (d, J = 7.8 Hz, 2H), 1.31 (d, J = 15.1 Hz, 6H), 1.25 (s, 12H), 0.88 (s, 3H). MS (m/z): 636 [M+].

#### DPP-BisTPA

100 mg (0.1570 mmol) of 2,5-didodecyl-3,6-di(thiophen-2-yl)-2,5-dihydropyrrolo[3,4-c]pyrrole-1,4-dione (DPP- alq), 253.69 mg (0.3425 mmol) of 4,4′-Bis[(4-bromophenyl) phenylamino]biphenyl, 65.09 mg (0.471 mmol) of potassium carbonate, 5 mg (0.022 mmol) of palladium acetate, 2.5 mL of N,N’ dimethylacetamide. The product was obtained as a dark blue-green powder (233.3 mg, 0.03959 mmol) in 84% yield. FTIR ν (cm^−1^): 3024 C-H, 2950 C-H, 2925 C-H, 1669 C=C, 1589 C=O, 1213 C=N, 1252 C-N, 701 C-S. ^1^H NMR (300 MHz, CDCl_3_) δ (ppm): 8.10 (s, 1H), 7.51 – 7.43 (m, 5H), 7.35 (d, J = 6.8 Hz, 2H), 7.29 – 7.24 (m, 6H), 7.15 – 7.12 (m, 4H), 7.10 – 7.05 (m, 6H), 7.01 (d, J = 2.5 Hz, 2H), 6.98 (d, J = 2.2 Hz, 2H), 4.09 (s, 2H), 2.14 – 2.03 (m, 2H), 1.33 – 1.14 (m, 18H), 0.95 (t, J = 7.4 Hz, 3H).

### Molecular design and theoretical calculations

Density functional theory (DFT) calculations have been performed using Gaussian 09w [66, 67]. To obtain the optimized structure and the frontier orbitals, B3LYP/ 6-31G (d, p) level of theory was used; To obtain the linear transitions of absorption and mission from the optimized molecular structures, TDDFT was used at the CAM-B3LYP/ 6-31 + G (d, p) level of theory with CPCM solvent model (chloroform).

### Characterization

NMR spectra were performed on a Varian 300 mHz spectrometer. Infrared spectra were performed with ATR using a Perkin Elmer spectrophotometer. Ultraviolet absorption (UV) spectra (in chloroform solution and film) were recorded using a Perkin Elmer Lambda XLS spectrometer Perkin Elmer Lambda XLS. The fluorescence spectra (in chloroform solution and film) were obtained using a Perkin-Elmer LS55 spectrophotometer. Cyclic voltammetry (CV) measurement was performed on a Princeton Applied Research Potentiostat/Galvanostat Model 263 A electrochemical instrument with a 3-electrode cell in a solution of 0.1 M tetrabutylammonium hexafluorophosphate (Bu_4_NPF_6_) in anhydrous acetonitrile at room temperature under nitrogen atmosphere with a scan rate of 50 mV/s. The working electrode was ITO with deposited **DPP-BisTPA** film, platinum wire as the auxiliary electrode and as reference electrode as an Ag/AgCl electrode. Ferrocene-ferrocenium (Fc/Fc + ) couple was chosen as the internal standard. For the determination of the electrochemical values of the ionization potential (Ip) and the electronic affinity (Ea) as reported in the literature [68]. The zeta potential of the dilute suspensions was measured with a Malvern Panalytical. The micrographs were obtained by Scanning Electron Microscope Quanta FEG-250 SEM instrument.

### Organic nanoparticle manufacturing

Aqueous suspensions were prepared by reprecipitation method: 1 mL of **DPP-BisTPA** dissolved in THF (1.13 × 10−4 M) was injected into an aqueous solution of cetyltrimethylammonium bromide (CTAB) (10 mL) at a concentration of 8 × 10−4 M under ultrasonic stirring for 30 min. The THF present was evaporated by bubbling in nitrogen and ultrasonic stirring for 30 min, the procedure was repeated three times.

### Uric acid degradation test

A mixture of phosphate buffer solutions with a pH of 7.25, uric acid and an aqueous suspension of the precipitated chromophore nanoparticles with final concentrations in the system of 7.5 × 10^−3^ M, 1 × 10^−4^ M and 1.5 × 10^−4^M, respectively, were used to prepare the aqueous systems. The solvent used was pharmaceutical-grade water for injections. The light source was an OEM model photodynamic therapy flashlight with simultaneous LEDs of 630, 660 and 850 nm with a power of 0.16 mW, placed 10 cm from the cell. Power was measured using a Newport model 2935T-C optical power meter. The degradation of uric acid is monitored by observing the reduction of the absorption band of uric acid at 290 nm using UV-Vis spectroscopy.

## Results and discussion

### Synthesis

**Fig. 1:**
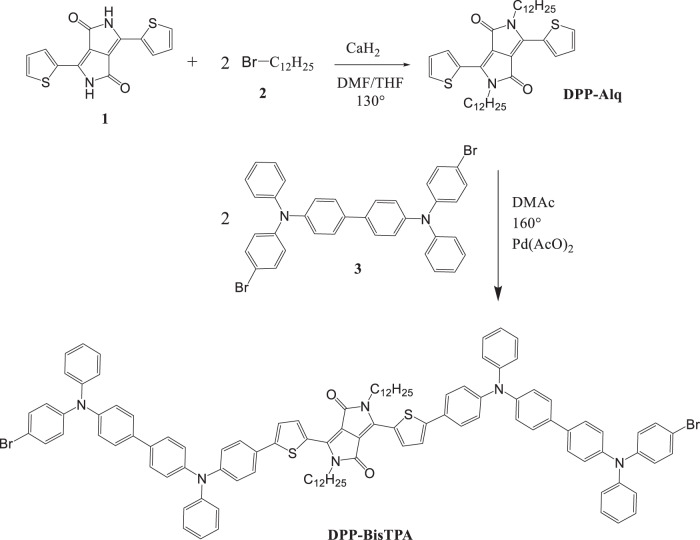
Synthesis of **DPP-BisTPA**

**DPP-BisTPA** was obtained according to the scheme, in Fig. 1. The product was purified obtaining 84% yield of the reaction, the product was characterized by FT-IR and ^1^H NMR spectroscopies.

**Fig. 2:**
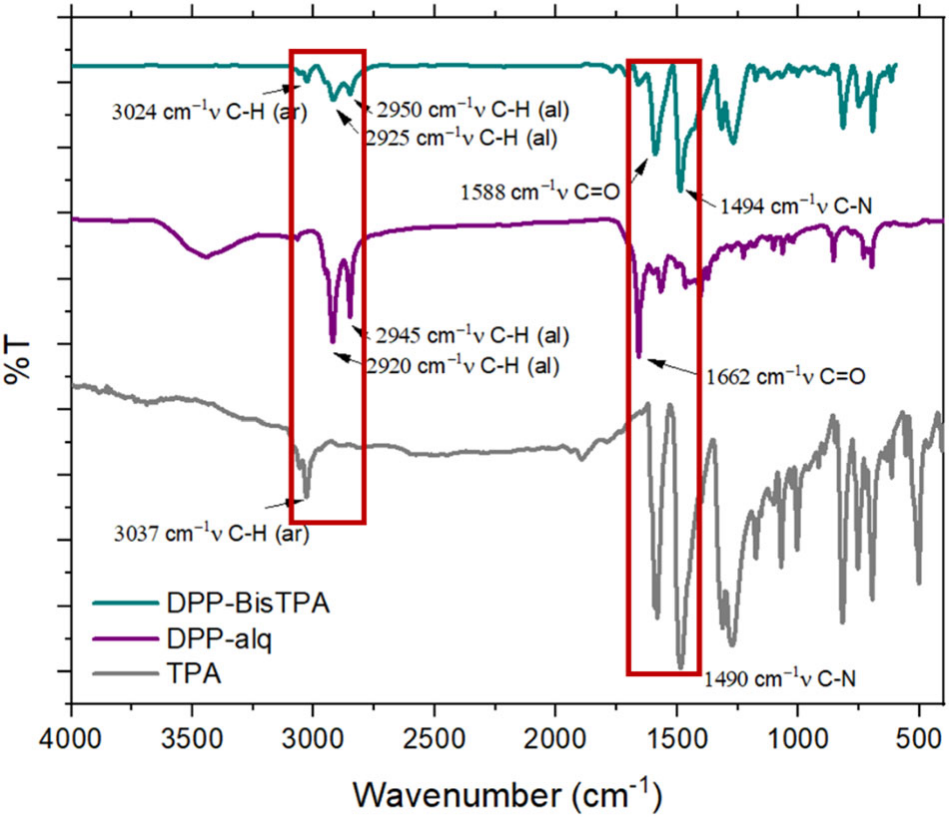
FTIR spectra of **DPP-BisTPA** and its raw materials

**Fig. 3:**
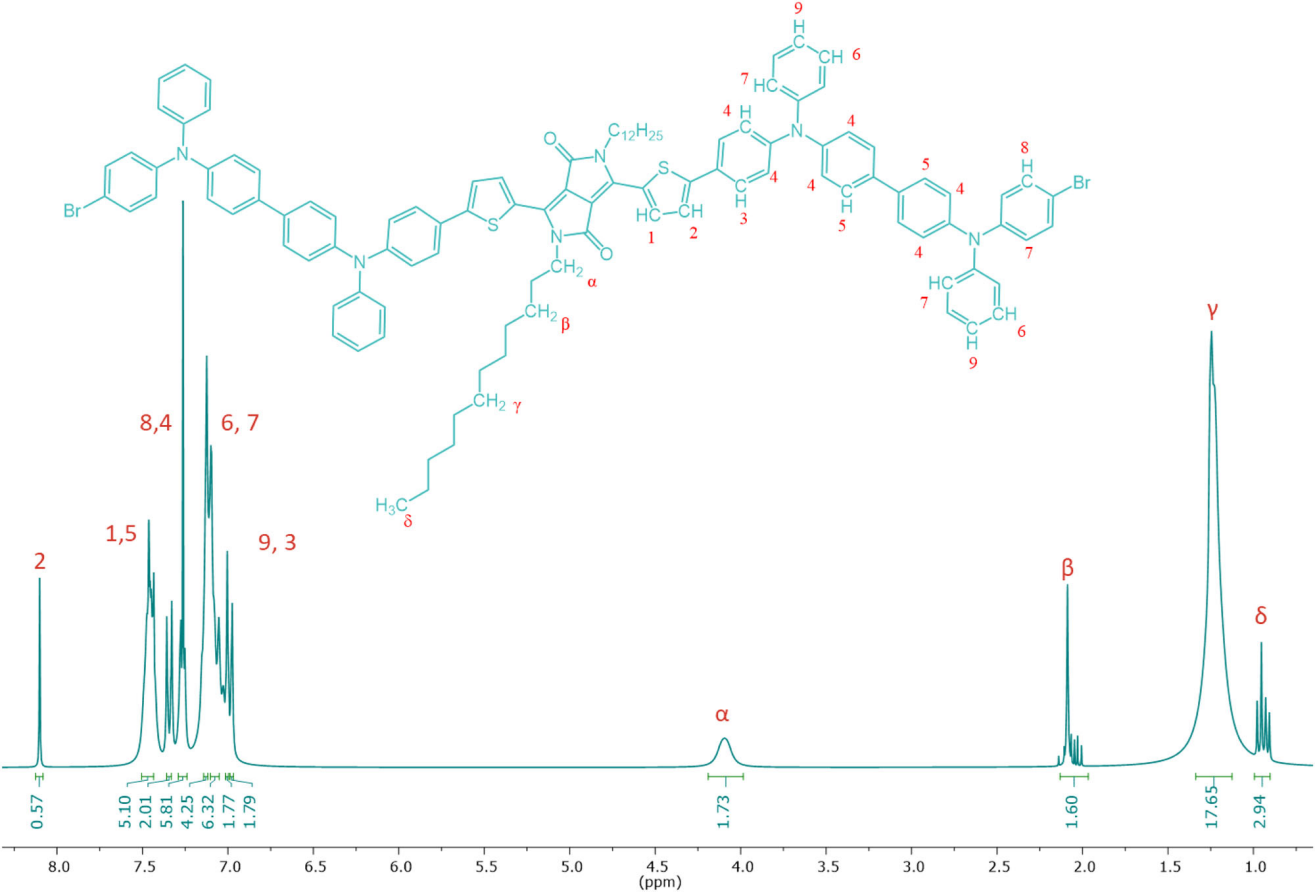
^1^H NMR spectra of **DPP-BisTPA**

Figure 2 shows the FTIR spectrum of **DPP-BisTPA** and its raw materials, in particular the appearance of the bands of the vibrational mode of the tension of the aromatic C-H bond at 3024 cm^−1^ provided by compound 3, and the corresponding bands of the aliphatic C-H bond of the chains bound to the nitrogen of DPP at 2950 and 2925 cm^−1^, as well as the vibrational mode at 1588 cm^−1^ of the C=O bond also of DPP. Figure 3 shows the NMR spectrum of **DPP-BisTPA** identifying the signals of the protons of the aliphatic chain bound to the nitrogen of the DPP ring and the signals of the protons of the aromatic rings, integrating the number of expected protons.

### DFT study

**Fig. 4:**
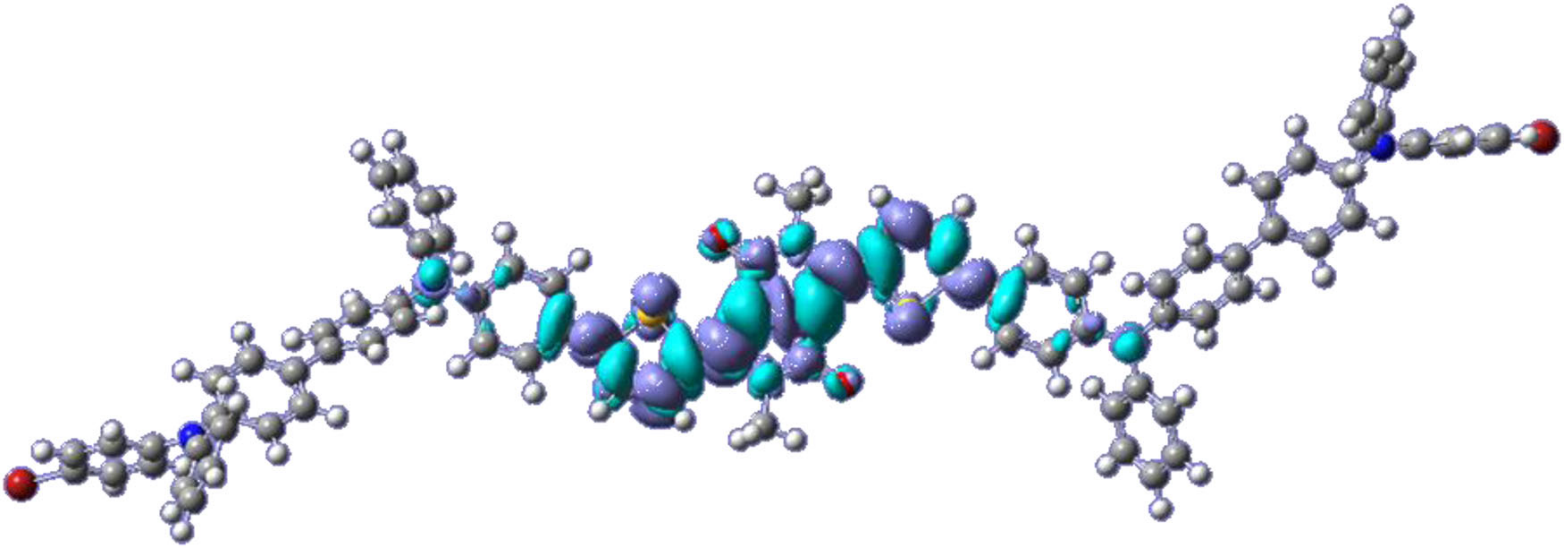
Isoimage of the difference density between the ground state and the excited state of DPP-BisTPA

The values of the energy levels of the HOMO and LUMO frontier orbitals were determined from the optimized structure of **DPP-BisTPA**. Figure 4 shows the isoimage of the difference between the excited and ground states of **DPP-BisTPA**. It can be seen that the charge transfer processes take place mainly in the central part of the molecule, which includes the DPP segment, the rings of the thiophene and the first phenyl of the triphenylamine, where planarity is lost.

**Fig. 5:**
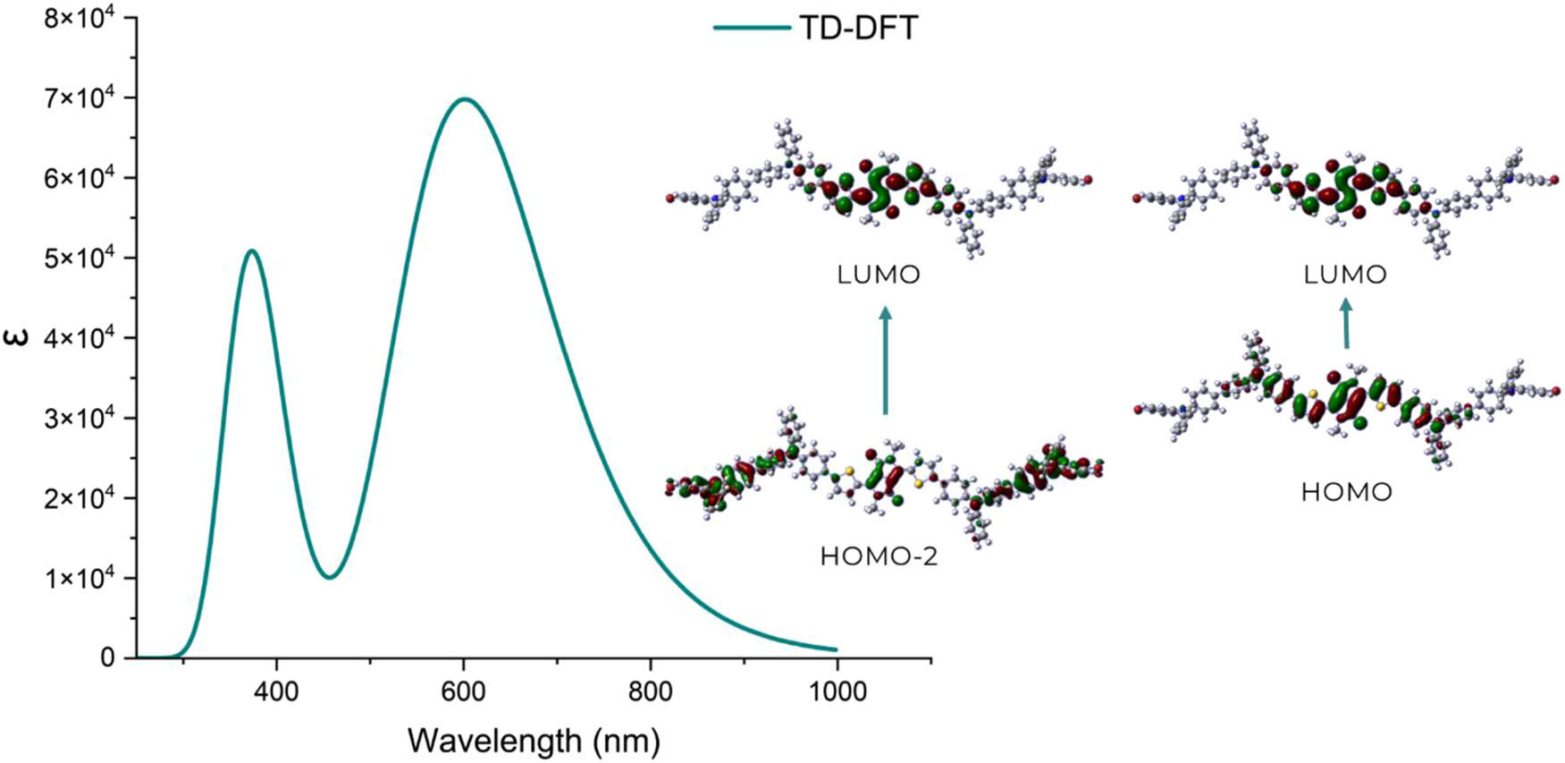
TDDFT absorption spectra obtained by CAM-B3LYP / 6-31 + (d, p)

Figure 5 shows the absorption spectra obtained from the TD-DFT calculation and in Table 1 the most probable electronic transitions. Based on the estimate, **DPP-BisTPA** presents two main electronic transitions, the first around 360 nm attributed to a transition between HOMO-2 and LUMO; and the second at 530 nm attributed to a typical transition from HOMO to LUMO energy levels [30, 69], resulting in a possible intramolecular charge transfer [70, 71]. Although the planarity is affected, there are charge transfer processes from the triphenylamine to the central receptor moiety of the DPP.

**Table 1:** Electronic transition data obtained by the CAM-B3LY´P/6-31 G (d, p)

λab (nm)	E(tr) (eV)	OS (ƒ)	MO/Character
530.12	2.3388	1.6670	H → L (0.88)
375.02	3.3061	0.0051	H-3 → L (0.17), H-1 → L (0.51), H → L + 1 (0.15)
360.54	3.4388	1.2844	H-4 → L (0.32), H-2 → L (0.37)

The energy gap between the first singlet and triplet excited state (Δ_S1→T1_) is important to be able to predict a possible intersystem crossing (ISC) that allows having a triplet excited state, a reduction in Δ_S1→T1_ favors ISC [72]. In Table 2 the estimated parameters are deposited, the Δ_S1→T1_ indicate a possible intersystem crossing, given the value of the triplet state, which could be the result of ICT processes.

**Table 2:** Estimated energy levels by CAM-B3LYP / 6-31 + (d, p)

HOMO (eV)	LUMO (eV)	Egap DFT (eV)	S_1_ (eV)	T_1_ (eV)	Δ_S1→T1_ (eV)
−4.59	−2.44	2.15	2.34	0.58	1.76

When the intersystem crossing is performed, the mechanism by which the ROS are produced can be estimated. There are two main mechanisms of the photodynamic reaction, either the excited triplet state of the photosensitizer can transfer electrons to the surrounding triplet state molecular oxygen to generate free radicals such as hydroperoxides, superoxides or hydroxyl radicals (type I), or it can transfer its energy to a ground state triplet oxygen to generate excited state singlet oxygen (^1^O_2_) (type II) when the transferred energy exceeds 0.98 eV [41, 73]. In each case, different types of cell death are promoted, depending on the intracellular position of the photosensitizer. Damage to mitochondria in the cell membrane and damage to lysosomes or the endoplasmic reticulum can lead to apoptosis, necrosis and autophagy, respectively [16]. Based on what has been described previously, **DPP-BisTPA** could generate ROS by mechanism I, it would generate free radicals because the estimated energy of its triplet state does not reach 0.98 eV.

### Optical characterization properties

**Fig. 6:**
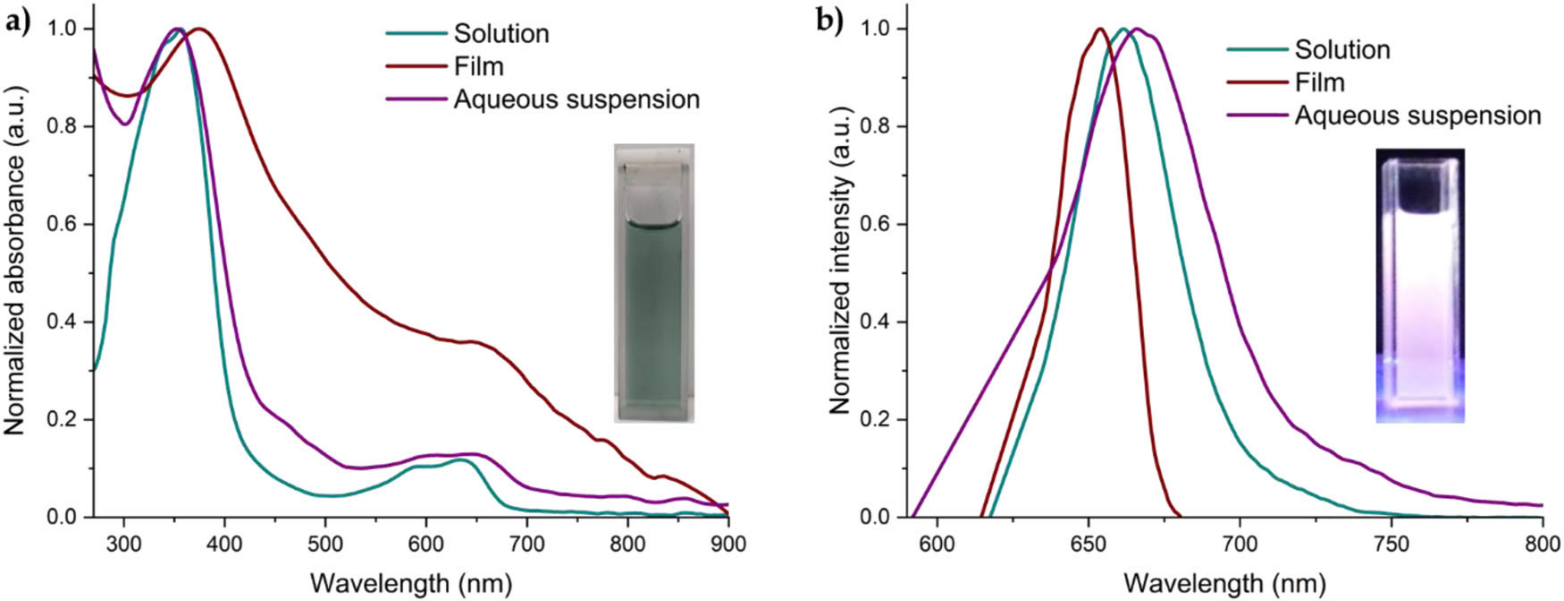
**a** Normalized absorption of **DPP-BisTPA** in THF solution, deposited on film and aqueous suspension of its NPOs, **b** normalized emission spectrum of DPP-BisTPA in THF solution, deposited on film and aqueous suspension of its NPOs

In Fig. 6 the absorption and emission spectra of **DPP-BisTPA** in solution, film and suspension are presented, two main electronic transitions can be observed, the most important being the most red-shifted. This is consistent with that estimated by TD-DFT, indicating possible charge transfer processes, which influence the value of the optical bandgap, which was estimated on film with a value of 1.53 eV, which is an excellent value for a conjugated system, entered within the range of organic semiconductors of interest for advanced applications. The maximum absorption wavelength of **DPP-BisTPA** processed as NPOs in aqueous suspension is 657 nm within the phototherapeutic window.

Table 3 shows the photophysical properties of **DPP-BisTPA**, the bandgap is around 1.5 eV, which is a very good value related to the region where it absorbs, the low fluorescence quantum yield indicates that the main process of relaxation is vibrational non-radiative, the intersystem crossover process is a process favored by vibrational relaxation. The reduction of the fluorescence quantum yield concerning the moiety of DPP precursor is attributed to an efficient ISC process [72, 74–76]. In this case, it may be due to the intramolecular rotation of the triphenylamine moieties. In the solid state (nanoparticles), the ISC process usually increases, which favors the generation of singlet oxygen, since a triplet state of the photosensitizer is required, which, when relaxed, stimulates cellular molecular oxygen [77].

**Table 3:** Photophysical properties of **DPP-BisTPA**

Photophysical property	Value
λ_max abs sol_ (nm)	635
Bandgap _opt sol_ (eV)	1.82
ε (L*mol^−1^*cm^−1^)	11600
λ_max abs film_ (nm)	657
Bandgap _opt film_ (eV)	1.53
λ_max abs NPOs_ (nm)	657
λ_max em sol_ (nm)	654
λ_max em film_ (nm)	662
Φ	0.01
Stokes Shiff	70

### Electrochemical characterization

**Fig. 7:**
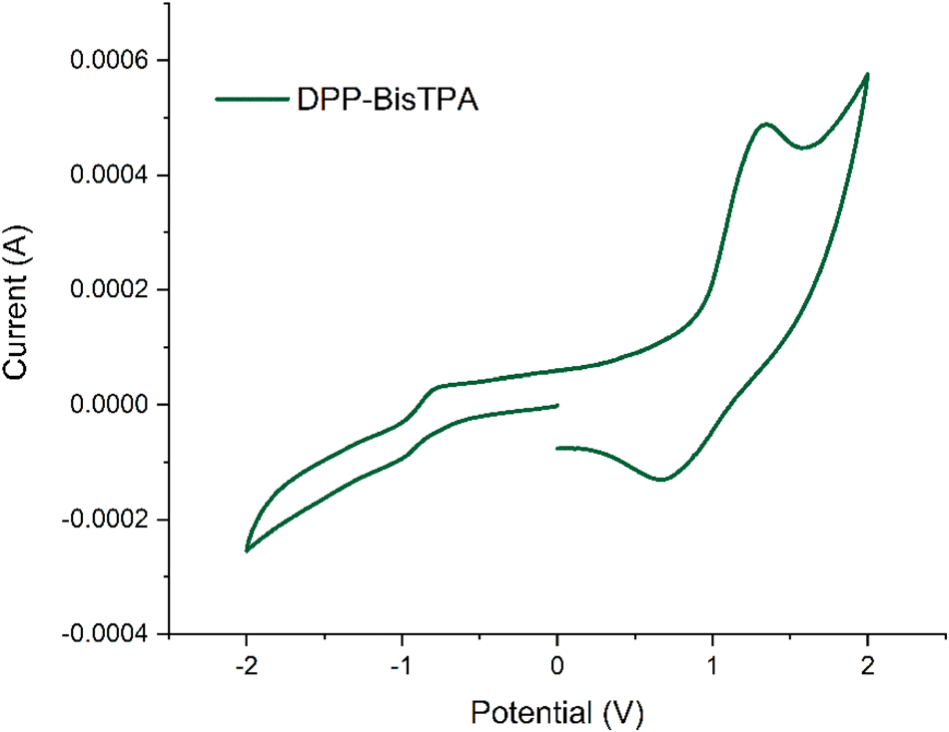
Voltammograms of **DPP-BisTPA**

Figure 7 shows the cyclic voltammogram of DPP-BisTPA, a quasi-reversible process is observed, with oxidation processes around 1 eV, and reduction processes also starting around −1eV, the frontier molecular orbitals were estimated, being −5.28 and 3.55 eV for HOMO and LUMO respectively, with an electrochemical bandgap of 1.73 eV, this corroborates the behavior as a possible organic semiconductor of **DPP-BisTPA**, as can be seen in Table 4.

**Table 4:** Electrochemical properties of DPP-BisTPA

Molecule	Onset reduction (V)	Onset oxidation (V)	IP HOMO (eV)	EA LUMO (eV)	Band Gap (eV)
DPP-BisTPA	0.92	−0.81	−5.28	−3.55	1.73

### Morphological characterization

**Fig. 8:**
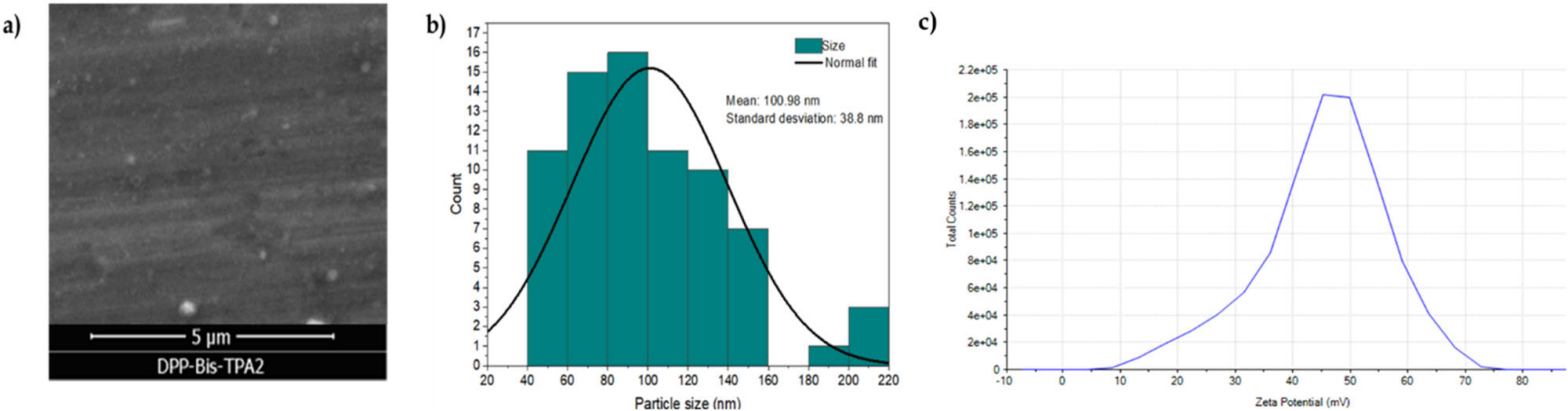
Micrograph and particle size distribution of **DPP-BisTPA**

Figure 8a presents a micrograph of the particles obtained in the reprecipitation process, as can be seen, the morphology of the reprecipitated particles is hemispherical with sizes around 100 nm (Fig. 8b), this size would allow internalization within the cancer cell to carry out photodynamic therapy [15].

Figure 8c shows the graph of potential Z, and Table 5 condenses the results of this study. Based on the results obtained, it can be affirmed that the **DPP-BisTPA** particles have good stability since the colloidal suspension presents a Z potential of 47.3 mV and it is reported that the nanoparticles with zeta potential values greater than ± 30 have high degrees of stability [78, 79]. Also, with electrophoretic mobility of 3.7 µmcm/Vs and conductivity of 0.149 ms/cm.

**Table 5:** Z potential analysis data

	Z Potential mV	Electrophoretic Mobility µmcm/Vs	Conductivity mS/cm
Mean	47.3	3.704	0.149
RSD %	3.86	3.87	0.388
Minimum	45.3	3.548	0.148
Maximum	48.9	3.83	0.149

### Uric acid test

**Fig. 9:**
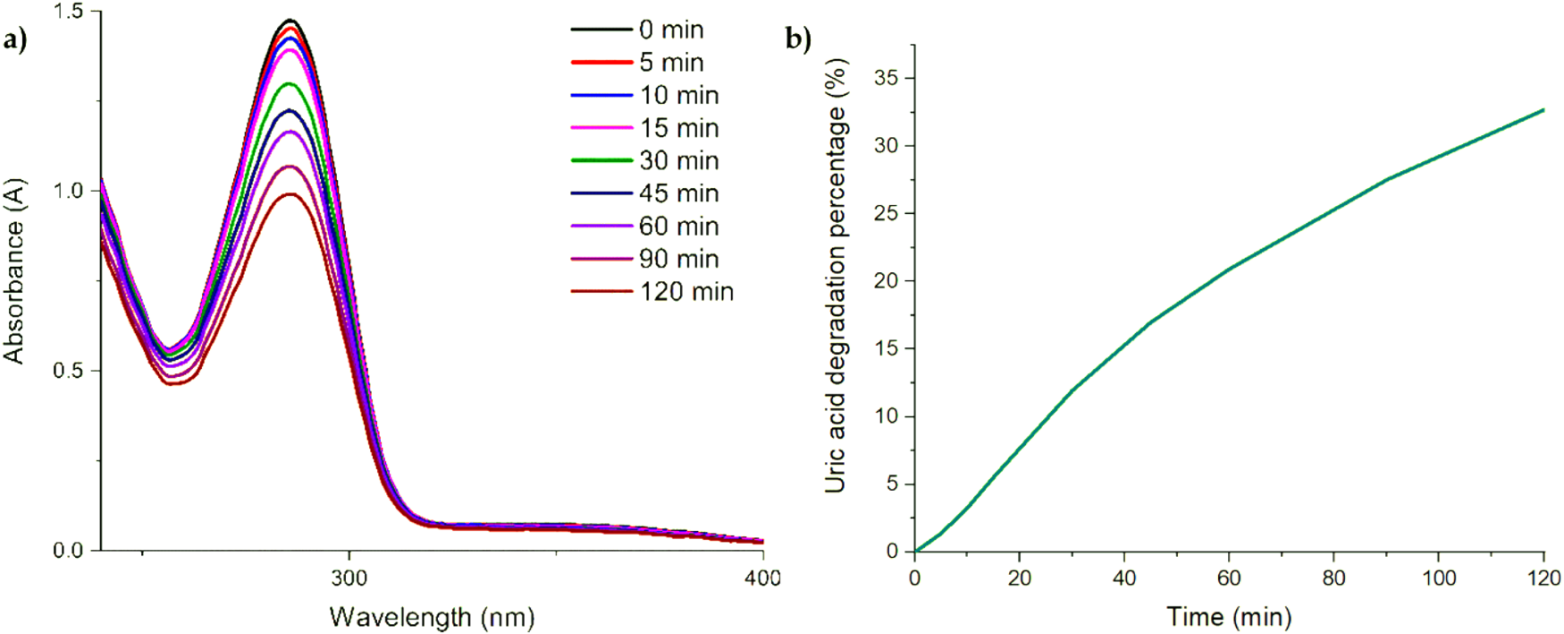
**a** Absorption spectra of uric acid degradation by ROS generation by **DPP-BisTPA**, **b** Kinetics of uric acid degradation in percentage

Figure 9 shows the uric acid degradation process through the action of **DPP-BisTPA**, for which a system within an absorption cell was used, it can be observed how the transition around 290 nm decreases with the step of time when in contact with the ONPs of **DPP-BisTPA** and under the irradiation of the phototherapy flashligth. Figure 9b shows the degradation kinetics. Table 6 summarizes the first kinetic parameter of uric acid degradation. It is worth mentioning that the stability of uric acid was tested under irradiation conditions in the buffer without the ONPs to verify that there is no photodegradation without the presence of ONPs of **DPP-BisTPA**. Likewise, tests were carried out previously with a white LED and a 532 nm green laser with a 3 mm beam waist and a power of 0.71 mW without having the effect of generating ROS, this is found in the supplementary material. This causes **DPP-BisTPA** to be activated only in the region of the phototherapeutic window using a dedicated PDT light source. The ONPs of **DPP-BisTPA in** exhibit a singlet oxygen generation yield of 4%. Possibly the energy gap between the first singlet excited state and the triplet is not short enough to make this process more efficient, which if carried out more efficiently would increase the generation of ROS. Although photosensitizers with yields between 2 and 4% have been reported [65, 80, 81], an attractive feature of DPP-BisTPA is that this activity as a photosensitizer has only been performed upon irradiation with light used in photodynamic therapy, thus avoiding processes with sources with wavelengths in the other regions of the visible spectrum.

**Table 6:** Kinetic parameters and singlet oxygen generation yield of DPP-BisTPA

Molecule	K_1_	ΦΔ
DPP-BisTPA	0.0036	0.04

## Conclusions

**DPP-BisTPA**, a molecule that exhibits good absorption properties, and bandgap, was synthesized. The ONPs obtained have hemispherical morphology with sizes around 100 nm, presenting absorption within the phototherapeutic window. **DPP-BisTPA** exhibits a ROS generation yield of 4%, this is possibly related to the absorption capacity of the molecule, since it is not so intense above 650 nm, since the relaxation processes are favored for it to be vibrational. unless the energy gap in the singlet and triplet excited states is somewhat distant. However, ROS generation takes place only when irradiated with a light source within the phototherapeutic window, which prevents the effects of photosensitivity to sunlight when treated with PDT. The ROS generation results are consistent with the computationally estimated. Therefore, **DPP-BisTPA** is a candidate to be evaluated as a photosensitizer in photodynamic therapy.

## Supplementary information

Supplementary Information

## Data Availability

All data generated or analysed during this study are included in this published article and its supplementary information file.
