# Correction to : Synthesis, and evaluation of photophysical properties of a potential DPP-derived photosensitizer for photodynamic therapy with D-A-D architecture

**DOI:** 10.1007/s10856-025-06873-8

**Published:** 2026-01-27

**Authors:** Vanessa Escalona Hernández, Itzia Irene Padilla-Martínez, Rosa Angeles Vázquez García, María Aurora Veloz Rodríguez, Oscar Javier Hernández-Ortiz

**Affiliations:** 1 Área Académica de Ciencias de la Tierra y Materiales, Carretera Pachuca-Tulancingo Km, Universidad Autónoma del Estado de Hidalgo (UAEH), 4.5.C.P. 42184. Ciudad del Conocimiento, Mineral de la Reforma, Hgo México; 2 Laboratorio de Química Supramolecular y Nanociencias de la Unidad Profesional Interdisciplinaria de Biotecnología del Instituto Politécnico Nacional, Av. Acueducto s/n Barrio la laguna Ticomán, Ciudad de México, 07340 México

Correction to: Journal of Materials Science: Materials in Medicine (2024) 35:11

10.1007/s10856-024-06776-0

In this article [1] the caption of Fig. 3 should read as “^1^H NMR spectra of DPP-BisTPA” instead of “RMN H^1^ spectra of DPP-BisTPA”. RMN should be replaced with NMR.

The caption of Fig. 8 should read as “Micrograph and particle size distribution of DPP-BisTPA” instead of “Micrografía y distribución de tamaño de partícula of DPP-BisTPA”.

“Micrografía y distribución de tamaño de partícula” should be replaced with “Micrograph and particle size distribution”.

The original article has been corrected.
