# Supplementary material for: Synthesis, and evaluation of photophysical properties of a potential DPP-derived photosensitizer for photodynamic therapy with D-A-D architecture
Source: J Mater Sci Mater Med. 2024 Feb 1;35(1):11. doi: 10.1007/s10856-024-06776-0 (PMC10834609; doi:10.1007/s10856-024-06776-0)
Supplement: Supplementary file 1 — Supplementary Information [file 10856_2024_6776_MOESM1_ESM.docx]

Synthesis, and evaluation of photophysical properties of a potential DPP-derived photosensitizer for photodynamic therapy with D-A-D architecture

V. Escalona Hernández^a^, Itzia Irene Padilla-Martínez^b*^, Rosa Ángeles Vázquez García^a^, María Aurora Veloz Rodríguez^a^, O. J. Hernández-Ortiz ^a,b*^

^a^ Universidad Autónoma del Estado de Hidalgo (UAEH). Área Académica de Ciencias de la Tierra y Materiales, Carretera Pachuca-Tulancingo Km. 4.5.C.P. 42184. Ciudad del Conocimiento, Mineral de la Reforma, Hgo., México.

^b^Laboratorio de Química Supramolecular y Nanociencias de la Unidad Profesional Interdisciplinaria de Biotecnología del Instituto Politécnico Nacional, Av. Acueducto s/n Barrio la laguna Ticomán, Ciudad de México, 07340, México.

* O.J Hernández-Ortiz: [ojavier.hdez@gmail.com](mailto:ojavier.hdez@gmail.com)

**Computational studies**

Electronic chemical potential (µ):

$\mu=H+L=\frac{1}{2}(-4.59-2.44)=-3.515eV$ eV

Electrofilicity (χ):

$$\chi=3.515 eV$$

Hardness (η)

$$\eta=L-H=-2.44+4.59 eV=2.15 eV$$

The electrophilicity index (ω)

$$\omega=\frac{\mu^{2}}{2\eta}$$

$$\omega=\frac{{(-3.515)}^{2}}{2*2.15 ev}=2.87 eV$$

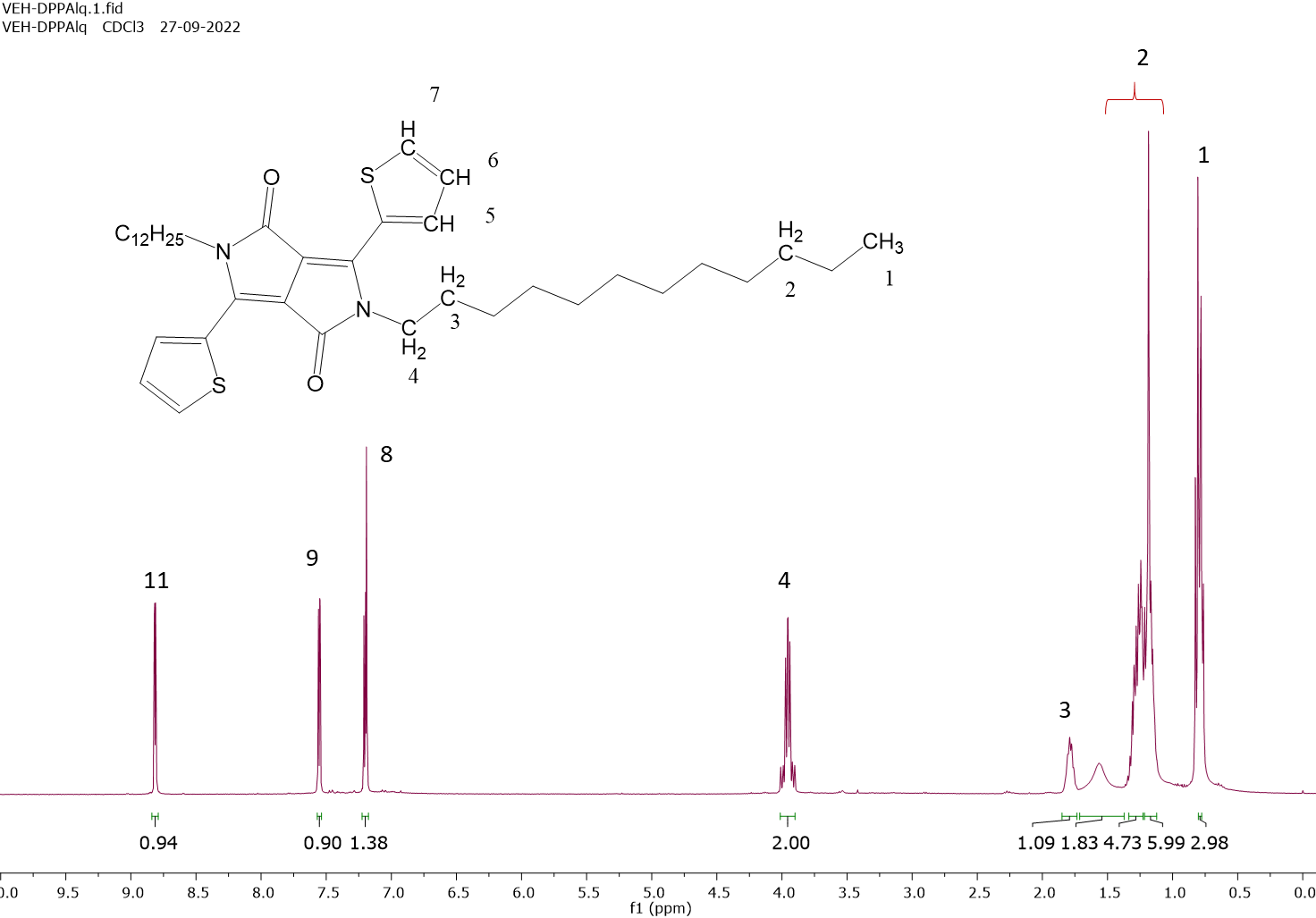


**S1.** ^1^H NMR spectra of DPP-alq


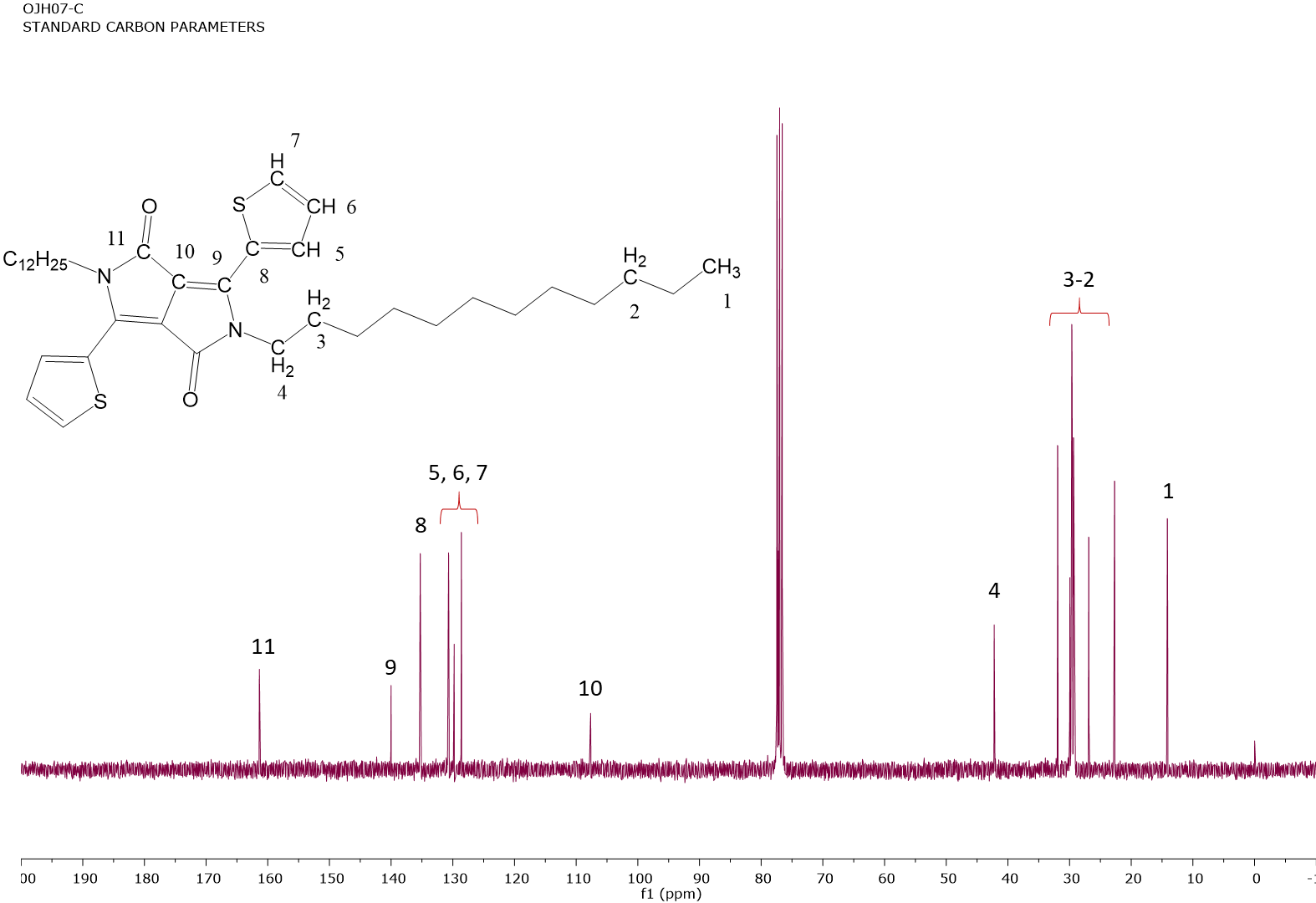


**S2.** ^13^C NMR spectra of DPP-alq

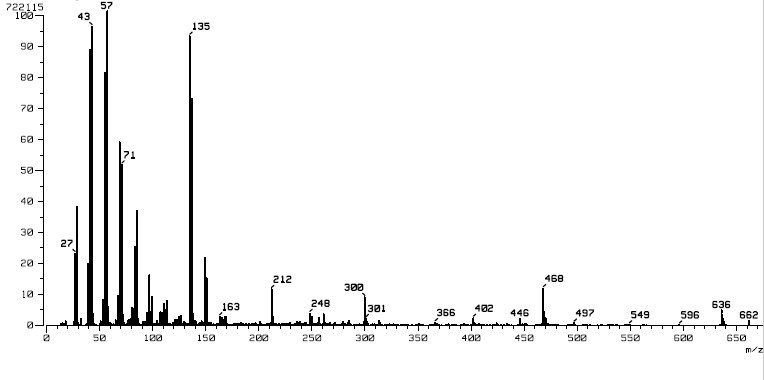


**S3.** Mass spectra of DPP-alq

**S4.** Determination of the molar extinction coefficient of DPP-BisTPA


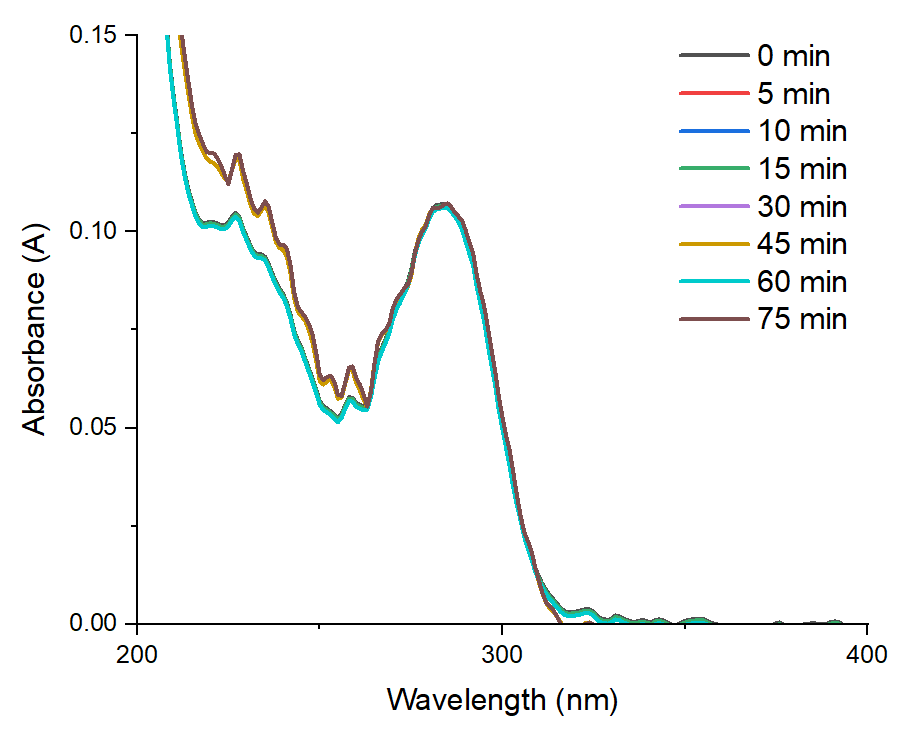


**S4.** Uric acid photostability test without the presence of DPP-BisTPA.


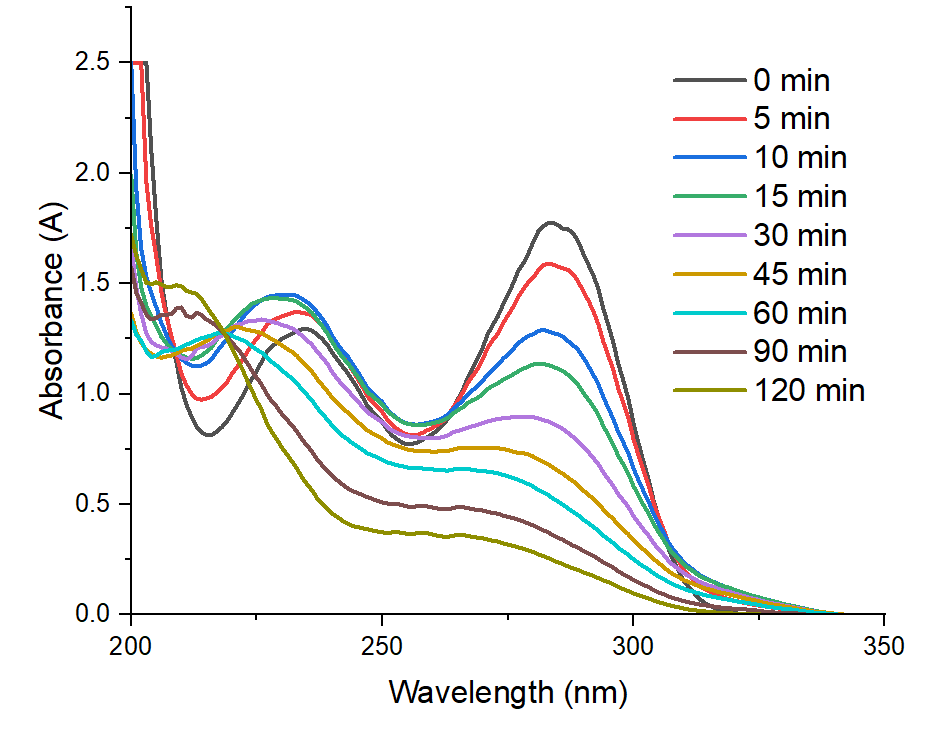


**S5.** Absorption spectra of uric acid degradation by ROS generation by Methylene blue, irradiated with a phototherapeutic flashligth.


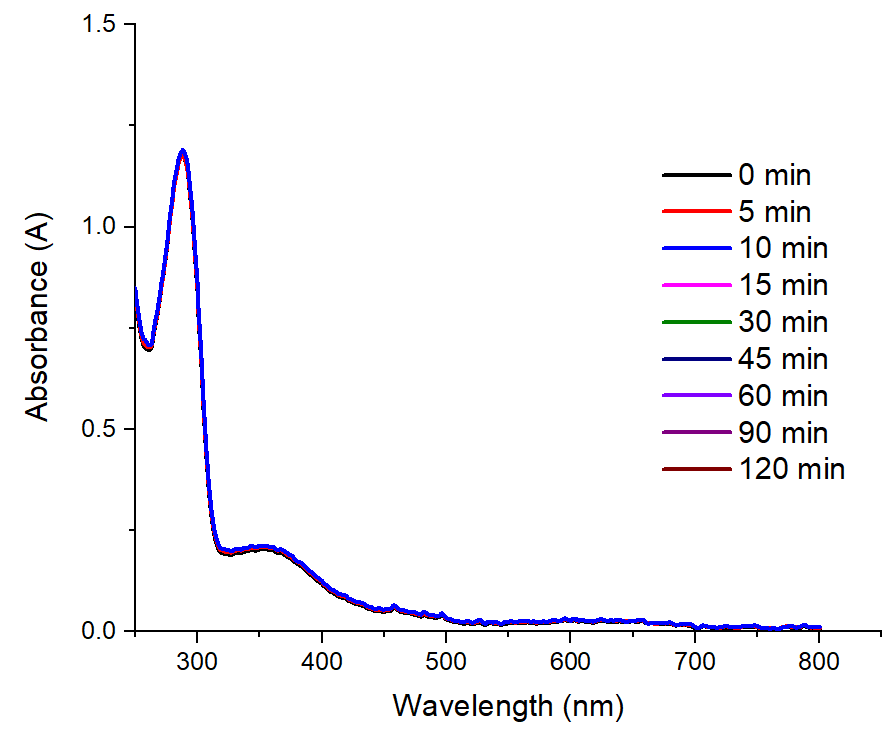


**S6**. Absorption spectra of uric acid degradation by ROS generation by DPP-BisTPA, irradiated with a commercial LED.


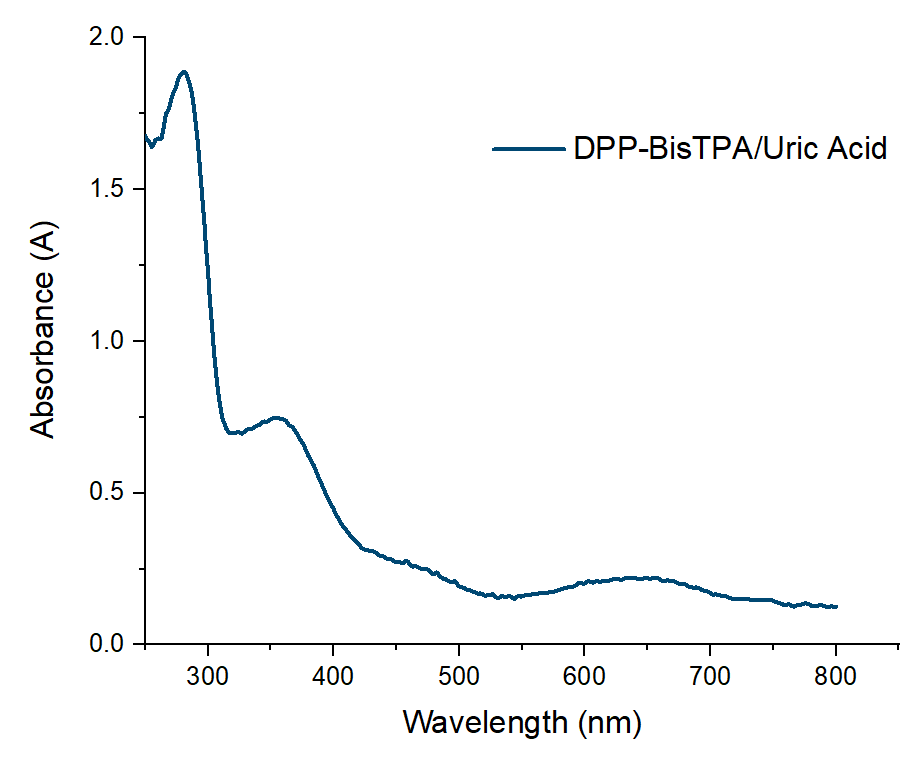


**S6**. Absorption spectrum of the monitored system consisting of uric acid, sodium phosphate and DPP-bisTPA


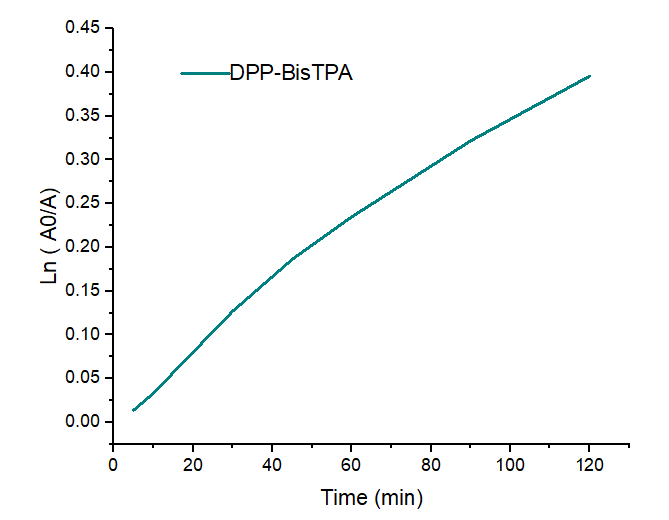


**S7**. Kinetics of uric acid degradation in the system consisting of uric acid, sodium phosphate and DPP-bisTPA and phototherapeutic flashlight as light source.
